# Short and Long-Term Soil Moisture Effects of Liana Removal in a Seasonally Moist Tropical Forest

**DOI:** 10.1371/journal.pone.0141891

**Published:** 2015-11-06

**Authors:** Joseph Pignatello Reid, Stefan A. Schnitzer, Jennifer S. Powers

**Affiliations:** 1 Department of Ecology, Evolution and Behavior, University of Minnesota, St. Paul, Minnesota, United States of America; 2 Department of Biological Sciences, Marquette University, Milwaukee, WI, 53201–1881, United States of America; 3 Smithsonian Tropical Research Institute, Balboa, República de Panamá; 4 Department of Plant Biology, University of Minnesota, St. Paul, Minnesota, United States of America; Tennessee State University, UNITED STATES

## Abstract

Lianas (woody vines) are particularly abundant in tropical forests, and their abundance is increasing in the neotropics. Lianas can compete intensely with trees for above- and belowground resources, including water. As tropical forests experience longer and more intense dry seasons, competition for water is likely to intensify. However, we lack an understanding of how liana abundance affects soil moisture and hence competition with trees for water in tropical forests. To address this critical knowledge gap, we conducted a large-scale liana removal experiment in a seasonal tropical moist forest in central Panama. We monitored shallow and deep soil moisture over the course of three years to assess the effects of lianas in eight 0.64 ha removal plots and eight control plots. Liana removal caused short-term effects in surface soils. Surface soils (10 cm depth) in removal plots dried more slowly during dry periods and accumulated water more slowly after rainfall events. These effects disappeared within four months of the removal treatment. In deeper soils (40 cm depth), liana removal resulted in a multi-year trend towards 5–25% higher soil moisture during the dry seasons with the largest significant effects occurring in the dry season of the third year following treatment. Liana removal did not affect surface soil temperature. Multiple and mutually occurring mechanisms may be responsible for the effects of liana removal on soil moisture, including competition with trees, and altered microclimate, and soil structure. These results indicate that lianas influence hydrologic processes, which may affect tree community dynamics and forest carbon cycling.

## Introduction

Intact tropical forests were once thought to be relatively resistant to anthropogenic changes in climate and atmospheric chemistry; however, these ecosystems now appear to be experiencing significant changes [1, 2]. Because tropical forests account for 40% of terrestrial net primary production and 25% of carbon stored in biomass in just 12% of land area [3], any change in tropical forest composition and function could have disproportionate effects globally.

One of the most apparent changes in tropical forests is the increase in liana abundance relative to trees, a pattern that is evident in multiple long-term studies from the neotropics [2, 4, 5]. Lianas (woody vines) are particularly abundant in tropical forests [6], commonly comprising 25% of the woody stems and more than 30% of the woody species [7]. Lianas are rooted in the soil and rely on the structure of their host trees to ascend to the canopy. Although liana stems typically account for less than 5% of the basal area or biomass of woody plants in tropical forests [8], their foliage can account for nearly 40% of leaf litter biomass [9, 10]. Lianas in tropical forests compete intensely with trees for light and belowground resources, reducing tree regeneration, fecundity, growth, and biomass in tropical forests [11–14]. While the mechanisms for increasing liana abundance are not yet known [2], lianas have the capacity to substantially alter tropical forest composition and function [10, 15].

Recent studies have quantified the effects of lianas on carbon storage in tropical forests. High liana abundance reduces the flux of carbon into terrestrial sinks [10], and displaces current sinks in tree biomass, decreasing overall forest carbon storage [15–17]. By contrast, we lack an understanding of how lianas affect other key global cycles—such as the hydrologic cycle in general and soil moisture in particular. Although many tropical forests receive considerably more precipitation than most temperate and boreal forests [18], rainfall and soil moisture is often distributed irregularly throughout the year and can limit tree growth and alter forest carbon cycles during seasonal or simulated experimental drought [19–21]. As tropical forests experience longer dry seasons due in part to climate change [22], trees may face even greater stress from low soil moisture if lianas are able to use soil moisture during the dry season [6, 23, 24]. Thus, the increase in liana abundance coupled with changing tropical rainfall regimes suggests that understanding how lianas affect soil moisture is an important component of predicting how tropical forests will respond to ongoing global change.

Lianas possess a number of traits that are distinctive from those of co-occurring trees and that may result in disproportionate effects on forest water and carbon cycles relative to liana biomass or basal area [25]. Lianas are reported to be deeply rooted [26], have longer xylem vessels [27] with larger diameters [24, 28, 29], higher maximum hydraulic conductivity [30], higher transpiration rates [31] and possess foliage with high N concentrations and hence photosynthetic rates [24, 32]. These generalizations about trait differences between lianas and trees appear relatively robust: however, they also yield a wide variety of predictions about how traits of individual lianas might scale to affect soil moisture. The potential for liana effects to vary seasonally and spatially through the soil profile further complicates predictions of liana effects on soil moisture.

The handful of prior studies that have experimentally investigated whether liana removal affects soil moisture levels in tropical forests have yielded contrasting results [11, 12, 33]. Using a target tree approach in a seasonally dry forest in Bolivia, researchers cut lianas from focal trees, and compared physiology and monthly gravimetric soil moisture data collected at two soil depths (about 10 cm and 100cm) [12]. Despite immediate and persistent differences in tree water status over the first several months of the experiment, there were no differences in soil moisture between trees with lianas and those in the liana removal treatment [12]. In another similar study in Bolivia, Pérez-Salicrup also did not detect differences in soil moisture measured with the same methods and measurement frequency where lianas were removed from small plots (900 m^2^) [11]. By contrast, in a large-scale experiment in northeastern Argentina where both lianas and bamboo were cut from 1 ha plots, soil water availability in the surface soil (5 cm depth) indexed by the filter paper method was higher in cut plots compared to control plots, but only in closed-canopy microsites and not under gaps [33].

Based on these empirical studies, it is clear that we presently lack sufficient understanding of the role of lianas in forest hydrology to predict whether liana removal should increase, decrease, or result in no net change in soil moisture. To fill in this critical knowledge gap, we used a large-scale, long-term, liana-removal experiment in a seasonal tropical moist forest in Panama to investigate spatial variability and temporal patterns of soil moisture, and the role of lianas in soil moisture dynamics. Due to the near impossibility of experimentally increasing the density of mature lianas in a forest, most tests of liana effects on tree physiology and soil moisture remove mature lianas from target trees or intact forest by cutting them at the base [14, 34–36]. We used this approach to compare soil moisture in patches of forests that contained lianas to those that lacked lianas. Our specific goals were to: 1) characterize seasonal patterns of soil moisture at different depths in the soil profile, and 2) determine if liana removal affected soil moisture and how any effects varied across seasons, years, and soil depth.

## Materials and Methods

### Site description

We conducted our study in a semi-deciduous, secondary, tropical moist forest on Gigante Peninsula (9.11 N, 79.85 W), Barro Colorado Nature Monument (BCNM), Republic of Panama. The Smithsonian Tropical Research Institute (STRI) has acted as the steward of BCNM for more than 50 years, and research conducted on the BCNM does not need a governmental (ANAM) permit. Our studies did not involve any protected or endangered species. The nearest meteorological station, on Barro Colorado Island, located 5.8 km to the northeast, receives 2700 mm of rainfall in an average year and temperatures average 27°C. Rainfall occurs throughout the year, but less than 5% typically falls in the dry season between mid-December and April. Most of the soils on the Gigante Peninsula are clay-rich, highly weathered Oxisols developed from andesite that overlies sedimentary material [37].

In 2008 we established sixteen 80 by 80 meter experimental plots (eight plots were randomly designated as liana removal and the remaining eight plots served as non-manipulated controls). We selected plot locations to minimize differences in topography and forest composition among plots. Plots were located in an approximately 60-year-old secondary forest located on the central plateau of Gigante Peninsula. In a pre-treatment census we found approximately 2000 liana stems (≥ 1 cm diameter) per ha and 3600 tree stems (≥ 1 cm diameter) per ha. Compared to other seasonal secondary tropical forests, liana abundances on the Gigante Peninsula are intermediate [38], suggesting that our results may be applicable to a wide range of tropical forests.

### Liana removal experiment

In April of 2011 we cut all liana stems in the removal plots. We cut stems at the base, but left the stems and crowns in place to avoid damaging trees and causing unnecessary side-effects [17, 35]. We controlled liana resprouting from cut stems in the removal plots by pruning cut lianas every three months for 2 years. We visited control plots for the same amount of time as removal plots to avoid a visitation effect in the removal plots.

### Soil moisture and temperature measurements

We installed time-domain reflectometry soil-moisture probes (EC5, Decagon, WA, USA) within the central 20 by 20 meters of all 16 plots approximately two weeks before cutting lianas. These sensors have an accuracy of ±3% VWC (volumetric water content), and we relied on factory calibration, which was appropriate for deploying the sensors in fine-textured mineral soil of Gigante Peninsula. To account for within plot heterogeneity we distributed two sets of five soil moistures probes across the north-south centerline of each plot (each set of five sensors shared a common logger). Within each sensor group, four probes were installed at 10 cm depth, where the bulk of the fine roots are located [39] resulting in eight shallow sensors per plot. These sensors were located at least 3 m apart. We placed the fifth sensor in each group at 40 cm depth to track changes in deeper water resulting in two deep sensors per plot. Fine root mass declines rapidly with depth in the soil profile on nearby Barro Colorado Island and other tropical forests [39], and the 40 cm depth criterion to represent deeper soil layers was based on known fine root distribution patterns. In June of 2013 we upgraded 80 of the 10 cm depth sensors to combination soil moisture and temperature probes (5TM, Decagon, WA, USA). We applied a calibration correction supplied by Decagon to the 2013 sensor values to account for an incorrect factory calibration. Loggers (EM50, Decagon, WA, USA) recorded measurements hourly and we downloaded data approximately quarterly.

### Statistical analyses

To estimate the effects of the liana cutting on soil moisture, we fit multi-level Bayesian models to soil moisture and temperature data. We designed the model to evaluate treatment effects at the plot level and to account for the grouping or pseudoreplication of loggers and probes within plots (equation 1).

$$\begin{array}{l} VWC_{treatment,day}\sim N(\mu_{probe,day},\sigma_{VWC,day})\\ \mu_{probe,day}\sim N(\mu_{logger,day},\sigma_{probe,day})\\ \mu_{logger,day}\sim N(\mu_{site,day},\sigma_{logger,day})\\ \mu_{plot,day}\sim N(\mu_{control,day}+\beta \times treatment,\sigma_{plot,day}) \end{array}$$

Where μ_control,day_ is the mean soil moisture at control sites on a given day, β_day_ is the effect of the removal treatment relative to controls on a given day and *N* is the Gaussian distribution.

We ran model simulations for at least 2000 iterations or until the simulations converged with good mixing and there were at least 200 effective simulations. We fit each day of our observational record separately (1055 total model fits). To account for pre-treatment differences in soil moisture between treatments, we subtracted the average soil moisture at each probe for the two rain-free days prior to the liana removal. We fit models to the adjusted soil moisture values, and report results in uncorrected units of volumetric water content (v v^-1^).

For parameters estimated in our models (control and removal soil moisture or temperature, and the difference between them) we report the mean of the posterior distribution and 95% credible intervals. We conclude that the removal mean is significantly different than the control mean if the 95% credible intervals for the treatment effect exclude zero. When reporting results across multiple single-day model runs, as when comparing or summarizing parameter estimates within a season, we report the median and the range for 95% of daily parameter means. We used Stan [40–42] and R [43] to fit the statistical models for the Stan model code for the soil moisture and temperature models (see S1 Appendix and S2 Appendix for model code).

## Results

After omitting observations due to faulty sensors or equipment failure, the final dataset contained 2,387,854 soil moisture observations from the 10 cm soil depth, 573,572 observations from the 40 cm soil depth, and 424,064 soil temperature observations (all at 10 cm). Median daily soil moisture values ranged from 16.7 and 44.6% in the top 10 cm and from 22.1 and 51.9% at 40 cm depth (2.5 and 97.5 percentiles respectively). Surface soil was drier than deeper soil 78% of the time, but was occasionally wetter in the wet season, presumably after recent heavy rainfall.

**Fig 1:**
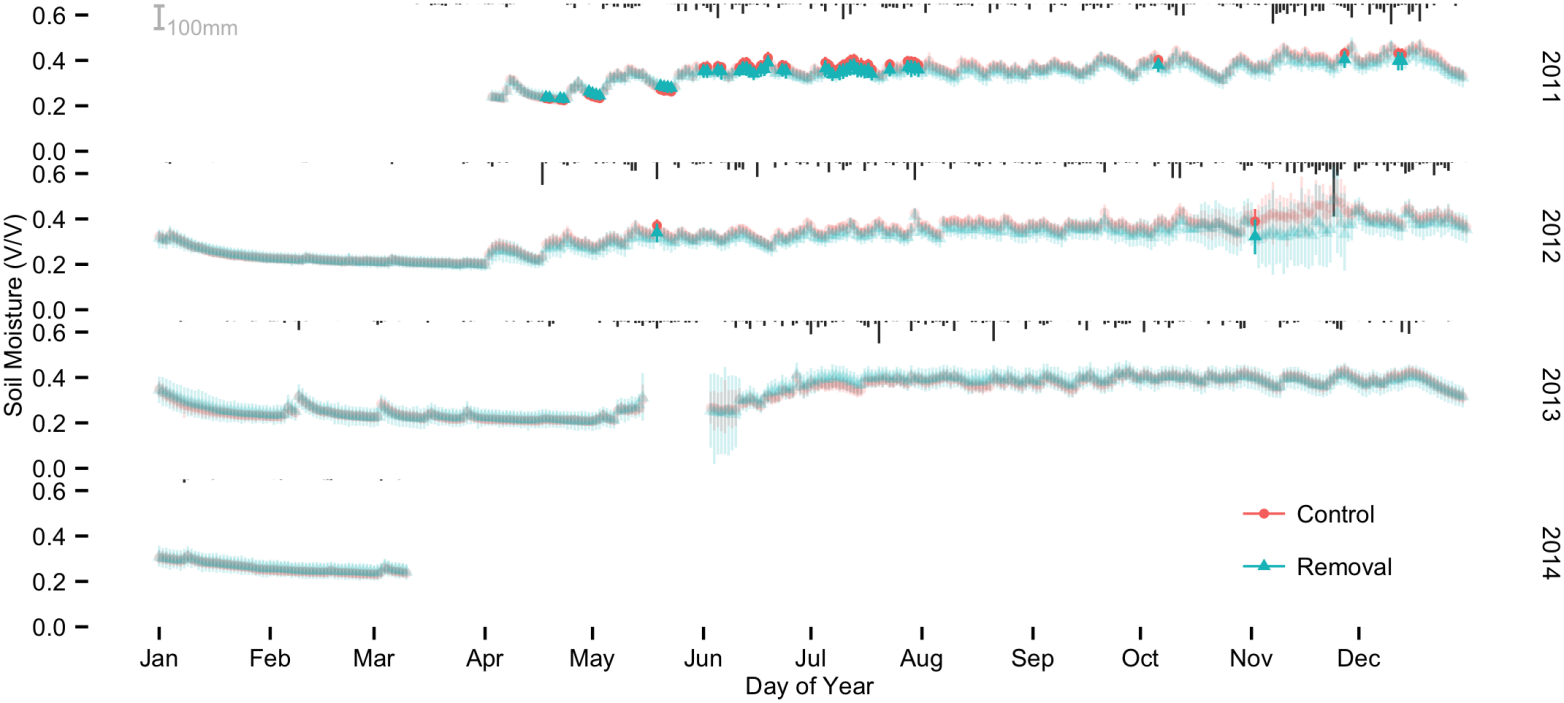
Treatment means and 95% credible intervals for 10 cm depth probes. Bold colors indicate dates with significant differences between treatments. Rainfall is in black at the top of each series.

**Fig 2:**
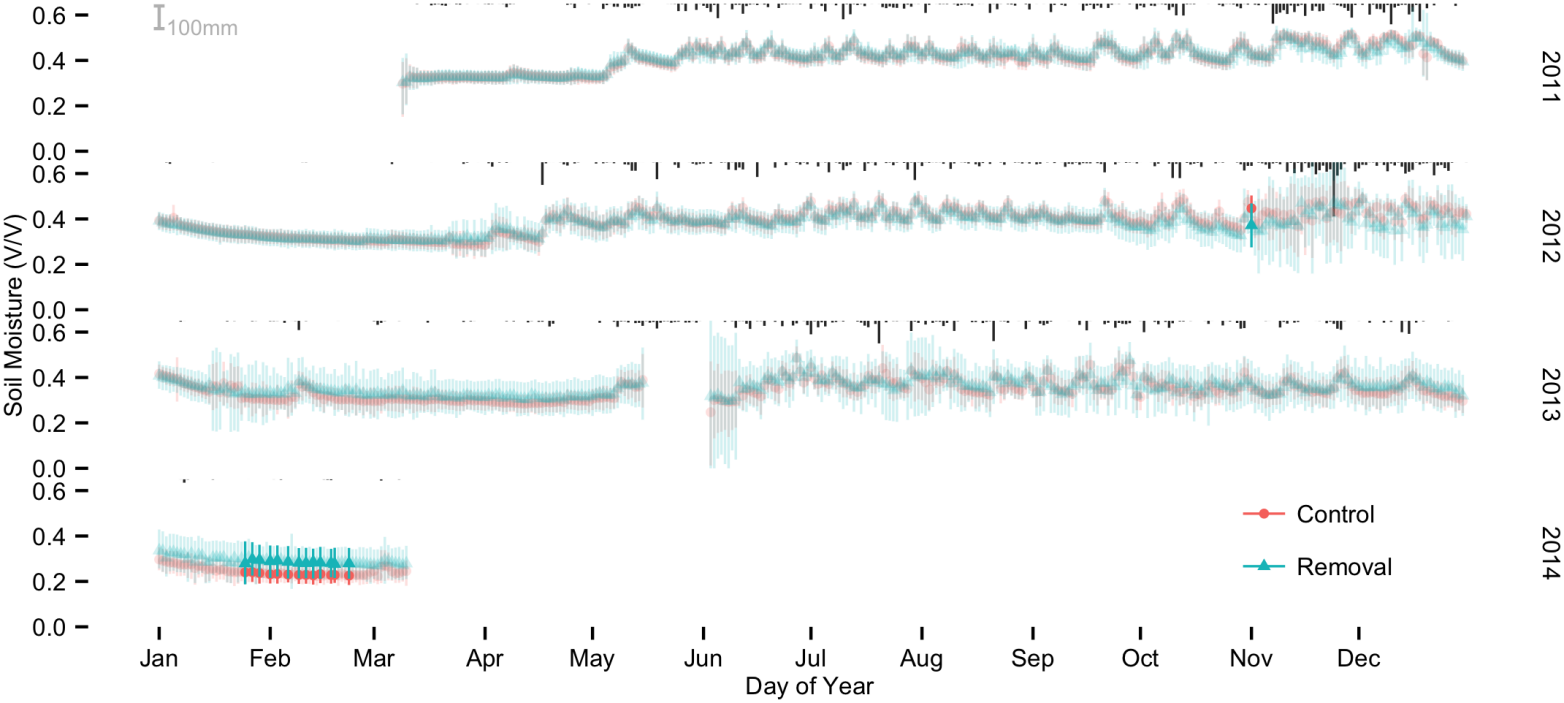
Treatment means and 95% credible intervals for 40 cm depth probes. Bold colors indicate dates with significant differences between treatments. Rainfall is in black at the top of each series.

**Fig 3:**
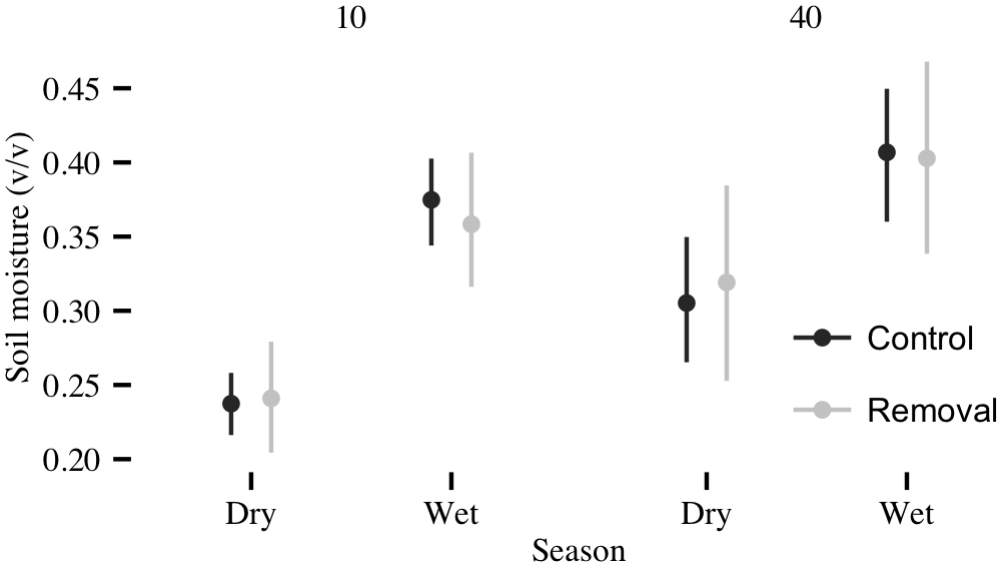
Median and 95% credible intervals of daily modeled soil moisture values by season and depth. Control plots in black, removal plots in grey.

Soil moisture varied consistently between seasons (Figs 1 and 2). During the dry seasons, soil moisture at the 10 cm depth dropped in an exponential-like fashion, but never fell below 20%. Soil moisture at 40 cm also dropped in an exponential fashion during the dry seasons, but typically decreased at a slower rate than at 10 cm. With the onset of the wet seasons (typically late April to early May), soil moisture increased rapidly. Median seasonal soil moisture increased from dry to wet seasons by 54% (from 0.24 to 0.37 v v^-1^) at 10 cm depth and 30% (from 0.31 to 0.40 v v^-1^) at 40 cm depth (Fig 3).

### Surface soil (10 cm)

**Fig 4:**
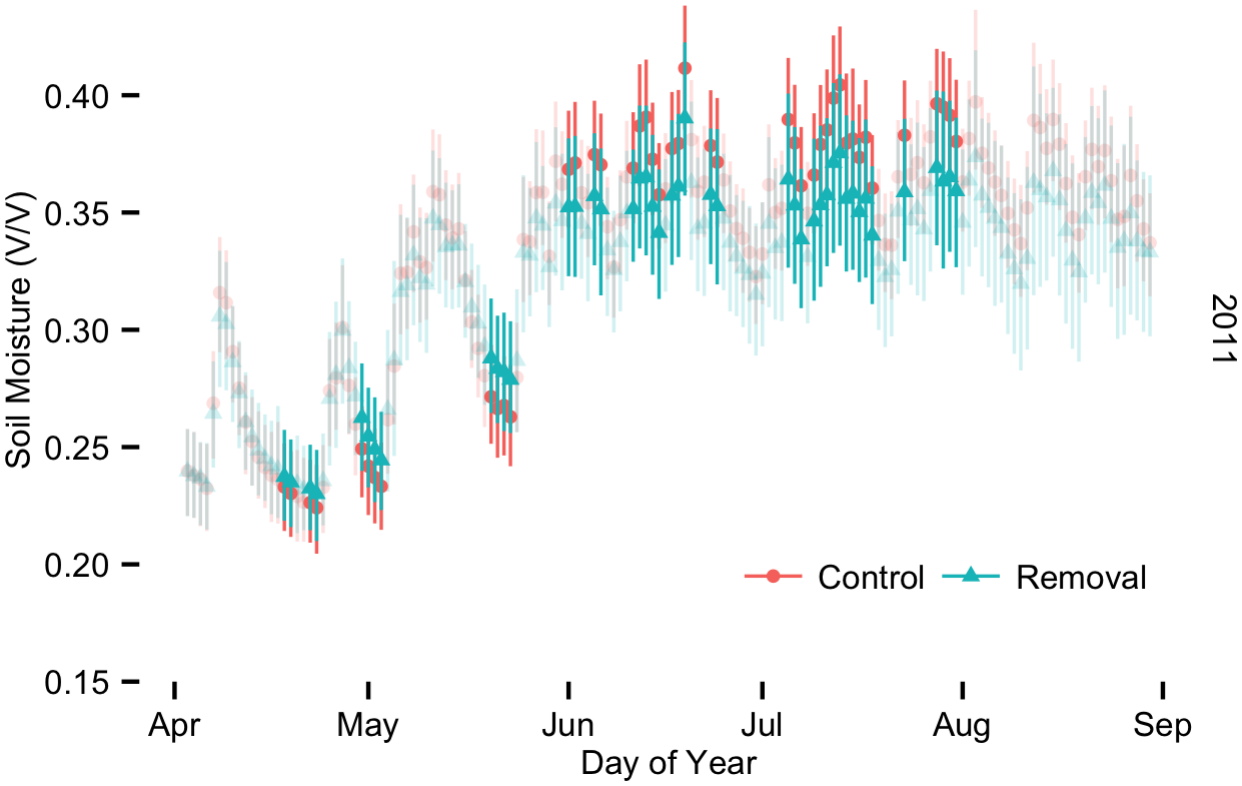
Soil moisture at 10cm during the first months of the experiment.

At shallow depths (10 cm) we observed two types of effects during the first five months after liana removal (May–August, including the transition into and first months of the wet season; Fig 4). First, removal plots dried more slowly than control plots during runs of at least seven dry days between rainfall of at least 2 mm. The difference in drying rates resulted in up to 6.5% higher soil moisture in the removal plots, which were statistically significant differences for some of the daily comparisons. Second, removal plots accumulated shallow soil moisture more slowly with the onset of the first post-cutting wet season (June–August), resulting in as much as 8% lower soil moisture in the removal plots during this period (also statistically significant for many of the daily comparisons). Liana removal thus resulted in a lower range of soil moisture values between the end of the dry season and the first months of the wet season.

During most (94%) of the wet season of 2011 (approximately June–December), which began shortly after liana cutting, liana removal resulted in a median value of 4% lower soil moisture than controls, but these values were not significant. Removal plots were also about 6% (median) drier than controls during 99% of the 2012 wet season. Despite somewhat drier soils in removal plots, both removal and control soils dried down to nearly identical soil moisture within weeks of the beginning of the dry season of 2012. From the beginning of the experimental manipulation throughout 2012, liana removal resulted in generally drier soil in the wet seasons and no difference between control and removal plots in the dry seasons, but again, few of these differences were statistically significant. Beginning in 2013, we found no significant liana removal effects. In summary, in the first four months following liana removal, we observed strong and consistent differences in surface soil moisture between removal and control plots, wherein the removal plots were first wetter and then drier relative to controls. However, in more than 800 days after the first four months we observed only 8 scattered days with treatment differences greater than the 95% posterior credible intervals, indicating that the initial effects of liana removal on soil moisture did not persist past the first season post-cutting.

### Deeper soil (40 cm)

**Fig 5:**
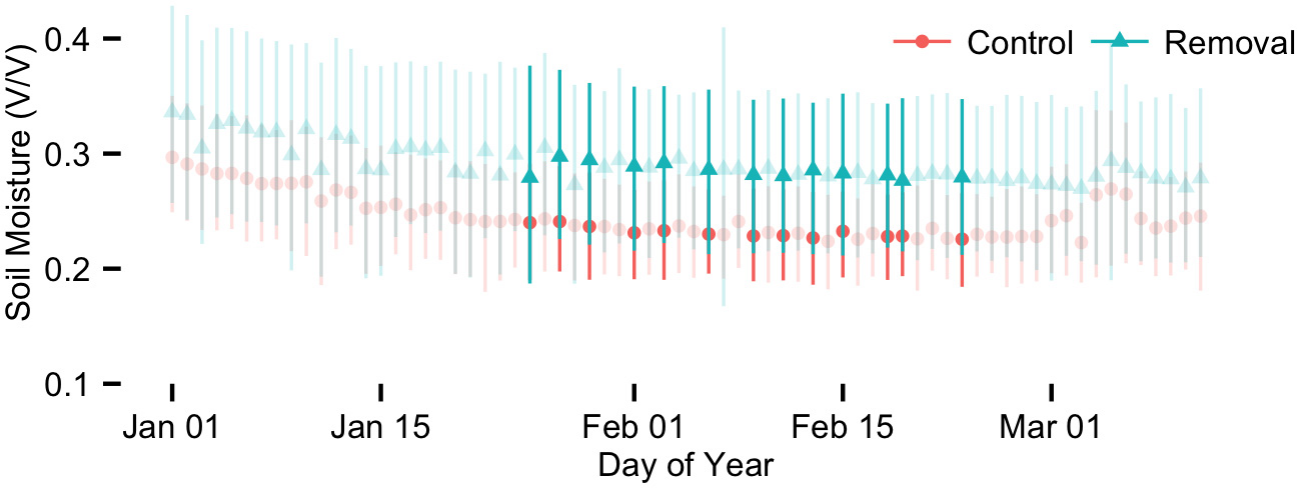
Soil moisture at 40 cm during the 2014 dry season.

**Fig 6:**
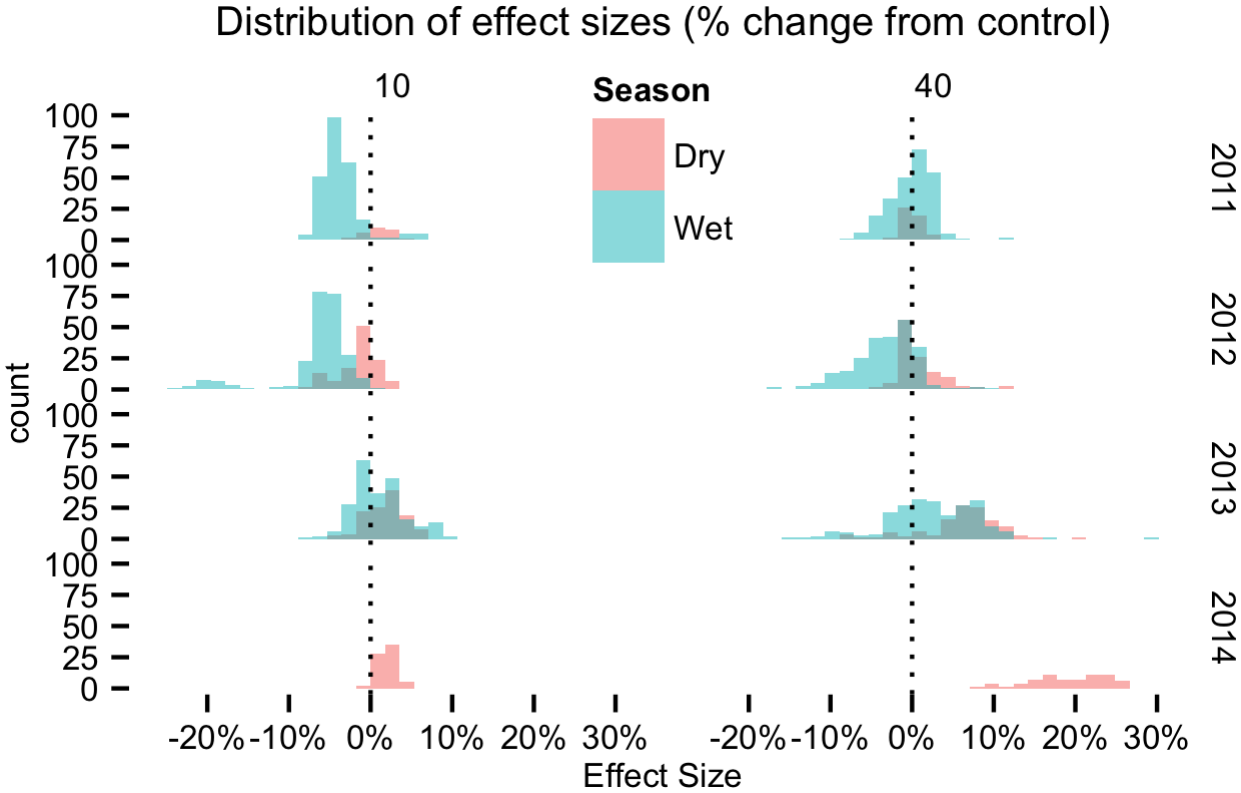
Histogram of relative effect size grouped by season and year for 10 and 40 cm soil depths. Days with higher soil moisture in removal plots relative to control plots appear to the right of the vertical dashed line. Distributions around zero indicate no consistent effect of liana removal. There are no trends across years in relative differences between the treatments during wet seasons at either depth. Soil moisture at 40 cm during dry seasons appears to trend toward consistently wetter in removal plots than the controls.

Visual assessment indicated that soil moisture was generally wetter at the 40 cm depth in the soil profile compared to surface soil, and showed less pronounced seasonal trends than surface soil (Fig 2). We observed no treatment effects outside the 95% posterior credible intervals except during the dry season of 2014. During the 2013 and 2014 dry seasons, soil moisture at 40 cm decreased more slowly in the absence of lianas. This difference in drying rates contributed to significantly wetter soil in removal plots during 13 days of the 2014 dry season and may indicate the beginning of a long-term treatment effect of liana removal on deep soil moisture. The significant treatment effects occurred in February as part of a dry season trend toward 5–25% greater soil moisture in plots without lianas (Figs 5 and 6).

### Soil temperature

Liana removal had a small and insignificant effect on surface soil temperature during the 2013 wet season and following dry season. Soils were consistently but not significantly warmer in the absence of lianas (mean difference = 0.073, range 0.00–0.23°C). We interpret this small difference to mean that the removal treatment did not affect soil surface evaporation two years into the experiment.

## Discussion

As lianas increase in abundance in tropical forests and these systems experience more variable and perhaps lower rainfall regimes, there is an urgent need to understand how lianas affect ecosystem-level processes, biogeochemical cycles, and forest hydrology. Predictions about how liana removal should impact soil moisture based on extrapolating liana traits from the individual to the community and ecosystem scale yield the entire gamut of potential responses, and thus complicate our ability to forecast how changing liana communities may affect tropical forests. Thus, empirical data from large-scale experiments such as ours are a useful starting point for constraining the range of future potential effects of increasing liana abundances on forest hydrology.

In our liana removal experiment, most of the significant responses we detected occurred within 4 months of liana cutting, and included both increases and decreases in surface soil moisture in removal plots relative to unmanipulated plots. We also found significantly wetter soils during the 2014 dry season at 40 cm depth. Our successful detection of significant differences between treatments gives us confidence that our methods were sufficient to quantify changes in soil moisture as a consequence of liana removal. Below we discuss potential mechanisms that might account for the observed patterns, the relationship between our study and prior work, and the implications for future tropical forests with increased abundances of lianas.

### Patterns and potential mechanisms

Soil moisture is assumed to reflect the net sum of processes including interception of rainfall, throughfall, infiltration, and root uptake [44–46]. Lianas may affect a number of these processes, and there are a number of potential outcomes of experimental liana removal on soil moisture. Furthermore, different mechanisms might result in similar patterns, and these patterns may change seasonally or with depth in the soil profile. First, no net change in soil moisture following liana removal may indicate that lianas do not affect soil moisture (at the depths observed). Alternatively, lianas may have a large effect on soil moisture; however, trees may completely compensate for this effect by rapidly using all of the liberated soil water, resulting in no net change. For example, sapflow in trees in the removal plots increased by ~60% immediately following removal compared to trees in the control plots [47]. It is also possible abiotic mechanisms such as either lower interception or increased evaporation from the surface soil that accompany opening up of the canopy affect soil moisture. Last, no change in soil moisture could also be found if the methods were insufficient to detect changes. Because we detected differences in soil moisture between control and removal plots, the latter possibility is unlikely.

### Surface soil

In surface soils our strongest results occurred immediately following liana removal and included a 6.5% increase in soil moisture in liana removal plots during the two months following liana removal, followed by a 5–8% decrease in soil moisture in removal plots relative to control plots for about two months, i.e. the amplitude of surface soil moisture variations was reduced in the liana removal plots. Increases in soil moisture following liana removal may be due to additional precipitation that reached the forest floor because of reduced leaf area and/or the inability of trees to take advantage of all of the additional soil water resources that were previously co-opted by lianas. In previous studies in this forest, we observed rapid sap flow increases in trees immediately following liana removal [47] during the dry season, which provides support for the hypothesis that trees are released from competition immediately following liana cutting and are able to take advantage of increased resource availability. Moreover, the increased soil moisture in liana removal plots during the first two months following liana cutting occurred only when overall soil moisture was declining after rainfall events (Fig 1). These patterns suggest that in liana removal plots during dry periods, there was not enough tree fine root mass to take full advantage of soil moisture, when soil moisture was presumably an important resource. Thus, it appears that the observed increase in sap flow immediately following liana removal [47] was insufficient to fully compensate for any soil moisture “liberated” through the liana removal.

We also observed a two-month period during the first wet season following liana removal where surface soils in removal plots were consistently drier than those in control plots (Fig 1). A number of mechanisms might decrease surface soil moisture following liana cutting. Removing lianas could dry surface soils if lianas increase surface soil moisture by hydraulic redistribution from deep soil, especially during the dry season, when lianas are believed to be more physiologically active than co-occurring trees [6]. Alternatively, if liana-tree competition is strongest for light, then removing lianas may increase tree growth [14, 47], resulting in lowered soil moisture in removal plots. This response is especially likely if trees were able to grow additional fine roots in the 3–4 months following liana cutting. Finally, altered microclimate could also decrease soil moisture through increased light penetration, warmer soil temperature, higher wind speeds or lower relative humidity; however, our soil temperature data do not support a microclimate effect. In summary, we observed both statistically significant increases and decreases in surface soil moisture in liana removal plots relative to control plots. These effects were limited to the first months of our three years of measurements and may indicate that trees in the removal plots adjusted rapidly to altered resource availability.

### Deeper soil

In deeper soils, liana removal resulted in consistently wetter dry season soil three years into the study (Figs 2, 5 and 6). The accumulation of wet season soil moisture could occur through reductions in dry season evaporative losses, reductions in deep soil water use, or changes in soil macropores as coarse liana roots decomposed. We expect any effect of microclimate to decrease over time (less drying) as trees recover and canopy leaf area increases. While we cannot rule out the contribution of microclimate effects in surface or deep soils, our temperature data suggest minimal effects. Alternatively, a prior study on BCI showed that lianas appear to use the same shallow water sources as trees during the beginning of the dry season and only switch to deep water sources at the end of the dry season (i.e. < 1 m depth) [48], thus, the observed increase in deep soil moisture during the dry season may reflect the reduction in water use after liana removal or perhaps changes in soil physical structure. Resolving whether these patterns persist into the future and what mechanisms account for them merits further research.

## Conclusions

Our dataset differs from previous efforts in terms of the: 1) large quantity of sensors deployed, 2) continuous measurements of soil moisture rather than snap-shot monthly values, 3) inclusion of soil temperature data, and 4) our multi-year record of soil moisture. In our large-scale, long-term experiment, liana removal had short-lived effects on surface soils in the first few months following removal. However, deep soil moisture increased in removal plots during the dry season three years after liana removal, suggesting that that the effects of lianas accumulated over time and that future research on this topic should include longer timescales. A wide array of mechanisms may be responsible for the changes we observed and future studies will allow us to estimate the importance of each potential mechanism. Although it is firmly established that lianas affect tropical forest community composition [13] and carbon cycling [10, 15], our results here demonstrate the potential effects of lianas on forest hydrologic processes.

## Supporting Information

S1 Appendix Stan model code for soil moisture model. (TXT)

S2 Appendix Stan model code for soil temperature model. (TXT)
